# Overcoming Career Plateaus: Enhancing Social Participation and Well-Being Among Middle-Aged and Older Adults

**DOI:** 10.1093/geroni/igaf122.2345

**Published:** 2025-12-31

**Authors:** Pei-Ying Yu, Ai-Tzu Li, Wen-Bing Gau

**Affiliations:** National Chung Cheng University, Taiwan (Republic of China); National Chung Cheng University, Minxiong, Chiayi, Taiwan (Republic of China); National Chung Cheng University, Minxiong, Chiayi, Taiwan (Republic of China)

## Abstract

Amid the global movement toward active and productive aging, there is a growing emphasis on promoting sustained employment among middle-aged and older adults to enhance social participation. In Taiwan, the combined effects of population aging and declining birth rates have led to continuous negative population growth and structural demographic shifts. These trends underscore the importance of developing and utilizing the middle-aged and older workforce, not only to enhance individual well-being but also to address labor demands in a super-aged society and foster positive social development. This study examines the career trajectories of middle-aged and older individuals, explores the challenges they encounter, and investigates career plateaus along with corresponding coping strategies. Using a qualitative research approach with purposive sampling(N = 12), semi-structured, in-depth interviews were conducted, examining three key perspectives. The thematic analysis revealed three key findings: (1) Middle-aged and older employees exhibit strengths such as workplace stability and professional expertise, with adaptability and continuous learning being crucial for career development; (2) Employment challenges are multifaceted, encompassing psychological well-being, job skills, and workplace interactions; (3) Effective strategies for overcoming career plateaus should integrate personal career planning with external support systems. The findings provide insights for adult education institutions, emphasizing the importance of continuous, personalized career development and goal setting, enhancing learning motivation, fostering an age-inclusive environment, and establishing support networks that facilitate intergenerational learning. For businesses, fostering a fair workplace culture, offering diverse and flexible employment options, and promoting lifelong learning mechanisms are crucial for building an inclusive and supportive work environment.

